# Fertility desire and associated factors among people on antiretroviral treatment at a public health facility in Hawassa city, Southern Ethiopia

**DOI:** 10.4314/ahs.v22i3.5

**Published:** 2022-09

**Authors:** Muche Argaw Eniyew, Yusuf Haji, Kelemu Abebe, Minchil Demelash, Yibeltal Mesfin, Aynamaw Embiale, Belay Amare

**Affiliations:** 1 Department of Midwifery, College of medicine and health sciences, Wolkite University, Wolkite, Ethiopia; 2 Department of Public health, College of medicine and health sciences, Hawassa University, Hawassa, Ethiopia; 3 Department of Midwifery, College of medicine and health sciences, Woliata sodo University, Woliata, Ethiopia; 4 Department of Midwifery, College of medicine and health sciences, Wachamo University, Wachamo, Ethiopia; 5 Department of Midwifery, College of medicine and health sciences, Wolkite University, Wolkite Ethiopia; 6 Department of Midwifery, College of medicine and health sciences, Madda Walabu University, Madda Walabu, Ethiopia; 7 Department of Midwifery, College of medicine and health sciences, Hawassa University, Hawassa, Ethiopia

**Keywords:** ART, Fertility desire, HIV/AIDS

## Abstract

**Background:**

Fertility desire is the plan of people to have a child or more children in the face of being diagnosed with HIV and plan to a commitment to implement the desire.

**Methods:**

An institutional-based cross-sectional study was conducted in Hawassa city public health facilities from May 09 -July 07/07/2019. Four hundred (400) study participants were selected using a simple random sampling technique. Data were collected by using interviewer-administered pre-tested structured questionnaires and chart review. The collected data were entered into EPI data version 3.1 software and then transported to SPSS version 20 for cleaning and data analysis. Bivariate and multivariate logistic regression was used to identify associated factors at p<0.05 was taken as a significant value with a 95% confidence level.

**Results:**

A total of 400 clients were included in the study giving a response rate of 97 %. The overall fertility desire was 53.6 %(95%CI: 48.7%, 58.2%). Age, sexual practice in the last six months and discussing reproductive health with ART providers were significantly associated with fertility desire. Younger age was positively associated with fertility desire, age group (18–29), [Adjust odds ratio = 5.75 95%CI (2.85, 11.57)] , age group(30–39), [Adjust odds ratio= 4.71 95%CI:(2.55, 8.71)] Sexual practice in the last six months [Adjust odds ratio = 3.00 95%CI(1.46 , 6.16)] and counseling reproductive health with ART provider[Adjust odds ratio = 3.10 95%CI:(1.86,5.15)]

**Conclusion:**

The prevalence of fertility desire in this study was higher than previous studies while factors associated with fertility desire were age, sexual practice in the last six months, and discussing reproductive health with ART providers.

## Introduction

Fertility desire is the plan of people to have a child or more children in the face of being diagnosed with HIV and plan to a commitment to implement that desire^1^. Globally many people on ART provide parenthood and family life because of improving their health condition. Increasing the quality of life for patients leads to a desire for fertility in people living with HIV/AIDS^2^. The coverage of ART for prevention from mother to child transmission was 57% in middle and low income. The fertility desires were increased due to easily accessibility and availability of ART drugs (UNAIDS 2012). After the initiation of ART, a patient's fertility preference increase among women that the prevalence of pregnancy was 22% in West Africa. The rate of pregnancy was 2.96 pregnancy /100 women-years of which the highest rate was in Burkin-Faso followed by Nigeria and Togo in West Africa countries^3^. ART used women who had higher pregnancy rates among HIV-infected women in sub-Saharan Africa. Increasing the incidence of pregnancy among HIV care and treatment programs in sub-Saharan Africa has an important indication for the health of women and their infants. Currently, married women in the reproductive age desired to another child were 18%, whereas 36% wanted to wait at least 2 years. Men and women had different childbearing limits, of which 27% and 37% wanted to limit their childbearing respectively. The ideal family size of women and men is 4.5% and 4.6% preferred child an average respectively. Overall the difference between the wanted fertility rate and fertility rate was one child, which implies that on average, women had one child more than they want^4^. Globally new acquiring of HIV was estimated at 430,000 children, of which over 90% of them through mother to child transmission. The risk of transmission was reduced from 45% to 2% if there were timely interventions during pregnancy, delivery, and breastfeeding 5. Worldwide the Magnitude of new HIV-infected people was 47%, of which, 95% were in Eastern Europe, Central Asia, the Middle East, and North Africa. In eastern and southern Africa new HIV-infected in adults was 710,000. The use of ART has increased the rate of pregnancy by 80%, and 33% were pregnant after 4 years follow -up in sub-Saharan Africa after starting ART. The increased overall incidence of pregnancy because of more than seven pregnancies per 100/year of observation within 4 years of follow-up after ART initiation in Sub-Saharan Africa^6^. Sub-Saharan Africa was most severely affected with nearly 1in every 20 adults (4.9%) living with HIV, of which 71% of adults and children were acquiring new HIV infection. One percent of adults were HIV positive in Eastern Europe, central Asia, and the Caribbean 7.

The fact that the majority of people living with HIV/AIDS are in reproductive age and heterosexual and mother to child transmission being major modes of transmission, a better understanding of the reproductive choice of people living with HIV/AIDS are important as the availability and accessibility of ART is improving in our country. This study is important in the prevalence of fertility desire and associated factors in HIV-positive males and females on ART in public health facilities in Hawassa city, Southern Ethiopia. To know the actual number of fertility desires and its associated factors among HIV/AIDS positive on ART is important to help infected individuals who desire to have children and such information can serve as improving for family planning services, accesses to unmet need for contraception, and PMTCT services. This is also important to generating knowledge, policy formulation, and program implantation in the dynamic world of HIV care providers in Ethiopia. Finally, the study will be significant by laying the foundation for further study and ultimately improving the knowledge of people about HIV/AIDS and reproductive desires.

## Methods and materials

### Study Area And Period

This study was done in four public health facilities at Hawassa city which is 273km located from Addis-Abeba, Ethiopia to the south. According to the city administer office report 300,025 total population was living.

### Study design & population

A facility-based cross-sectional study was conducted among people receiving ART follow-up care in the city. The study includes people with HIV with age 18–49 for women & 18–63 for men who had at least one visit to the ART unit.

### Sample size determination

The sample size was calculated using Open EPI version 3.03, by assuming the proportion of fertility desire 43%^8^ similar study in woliata sodo town, 95% of the level of confidence. The margin of error, d =5% Non-response rate 15%, and the exposure variable that significantly associated taken each variable, calculates the sample size for each variable. I compare the sample size that I gate in proportion and exposure variables that takes the largest sample size. I have got the proportion sample size is 348 and the largest sample size of the exposure variable is 276. I have used to the largest sample size of proportion of 348 and non-response rate take 15% = 348 x 15 % = 52.2 =52.The total sample size is 348+52=400.

### Sampling technique

**Figure 1:**
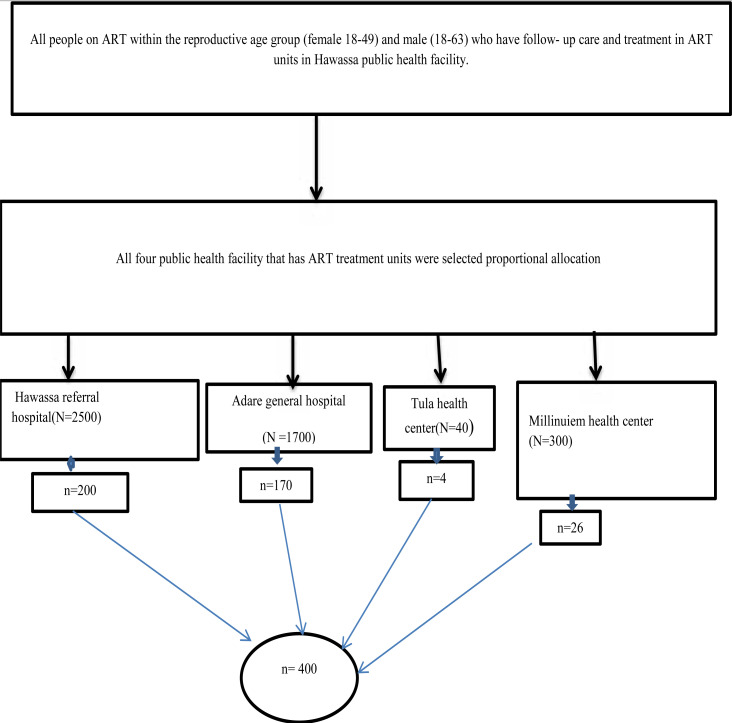
Schematic representation of sampling for fertility desire and associated factors among people on ART patients in Hawassa public health facilities, southern, Ethiopia, 2019

All four public health facility that provides care and treatment ART services were selected in the study and the number of samples needed for each public health facility was allocated based upon their patient load proportionally using sampling proportion. A sampling frame was prepared from the ART registration book based upon their appointment date and card number (that appointment date is the data collection period). Respondents were selected by simple random sampling methods by using a computer generating random samples from the prepared list of patients (figure1).

### Data collection technique and quality control

Structured interviewer-administered questionnaires were used that were adapted from Different literature related to the topic and modification in our context, which used to assess factors of fertility desire among ART patients. Data was collected through face-to-face interviews by pretested and interviewer-administered structured questionnaires among participants. The questioner was prepared in English and translated into the local language Amharic and finally back to English to maintain its consistency. Orientation was given for data collectors regarding the objective of the study, data collection tool, and procedure before the actual data collection. Moreover, the questioner was pre-test on 5 %(n=20) of the total samples in the Bushlo health center among ART patients who were not selected for actual data collection for clarity, simplicity, understandability, and coherency. Three clinical nurses and one BSC nurse who was working in each data collecting site were collected the data and two BSC nurse was recruited as supervisors who were working in Adare and Millennium health center. The interviewer was selected based on their work experience and ART training. Data quality was checked during data collection. Supervisors and principal investigators were closely following the data collection process. Questionnaires were checked daily for completeness. Data cleaning was done before data analysis by a computerized system.

### Data processing and analyses

Data were checked, entered into, and cleaned using EPI data version 3.1, and then exported to SPSS version 20 for further analysis. Descriptive analysis such as proportions, percentages, frequency, and distribution was employed. Binary logistic regression was done to examine the relationship between the proposed factors and a desire for fertility. Dependent variables assessed by 9 questions ranged from a 1–5 Likert scale that contains 1. Strongly disagree 2. Disagree 3. Neither agree nor disagree 4. Agree 5. Strongly agree then recoding after that computing variable adding them and calculate a mean score of 22.3 with a Cranach's alpha of 0.72 and dichotomies the dependent variables according to mean which above the mean have fertility desire(yes) and below the mean were have not fertility desire(no). Variables with a p-value of less than 0.2 in the bivariate analysis were re-entered into a multivariable analysis to identify variables independently associated with the desire for fertility. Enter method was used in logistic regression with model fit assessed based on the log-likelihood ratio test. Ninety-five (95%) CI with respective adjusted odds ratios (AOR) was used to assess the statistical significance of association among the variables and P-value <0.05 was considered as statistically significant.

## Results

Socio-demographic characteristics of study participants Out of 400, 388 participants were in the study with a response rate of, 97 percent. The mean (+SD) age of the study subjects were 33.74 ± (7.702). Among them, 248(63.9%) of participants were females and, 140(36.1%) were males. The majority age of the study participants 195(50.3%) was in the age group of 30–39. More ethnic participants in the study were Sidama 102(26.3%) and Amhara 80 (20.6%). Most of the study participants were lived in urban, 316(81.4%) whereas 72(18.6%) lived in rural areas. In the educational status of the participants of study 119(30.7%) were completed primary education followed by 94(20.4%) were completed secondary education. Concerning occupational status, 115(29.6%) were government employees 168(43.4%) were farmer, and others (unemployed, daily labor, and housewife) was 105(27.1%) (Table 1).

**Table 1:** Socio-demographic characteristics of participants on ART at Hawassa city, Southern Ethiopia, Ethiopia January 2019

Variables	Numbers	Percent
Sex		
Male	140	36.1
Female	248	63.9

Age		
18–29	105	27
30–39	195	50.3
>=40	88	22.7

Religion		
Protestant	208	53.6
Orthodox	147	37.9
Muslim	27	7
Catholic	6	1.5

Marital status		
Married	235	60.6
Single	48	12.4
Widowed	53	13.7
Divorced	52	12.3

Residence		
Urban	316	81.4
Rural	72	18.6

Educational level		
Can't read and write	56	14.4
Can read and write	63	16.2
Primary education	119	30.9
Secondary education	94	24.3
College and above	56	14.4

### Reproductive history and family planning of the study subjects

Three hundred thirty-four (86.1%) were having children, out of those 184(47.4) had 1–2 children and 150(38.7%) were having three and above children. One hundred sixty (41.2%), were birth after HIV diagnosis, among those 24(6.2%) were positive 5 (1.3%) were don't know their status, and 28(7.2%) children were lost related to HIV/AIDS. Two hundred nine (53.9%) used family planning among those 163(42%) were using short-acting family planning and the reason for choosing types of family planning 170(43.8%) was counseling from health professionals(Table 2).

**Table 2:** Reproductive history and family planning of participants on ART at Hawassa City, Southern Ethiopia, Ethiopia January 2019

Variables	Numbers	Percent
Have a child		
Yes	334	86.1%
No	54	13.9%
Number of children (n=334)		
1–2	184	47.4%
>=3	150	38.7%

Childbirth after diagnosis of HIV (n=334)		
Yes	160	41.2%
No	174	44.8%

HIV status of their children (n=160)		
Positive	24	6.2
Negative	131	33.8
Don't know	5	1.3

Child loss related to HIV		
Yes	28	7.2
No	360	92.8

Discuss-RH with ART provider		
Yes	174	44.8
No	214	55.2

Use of FP after HIV diagnosis		
Yes	209	53.9
No	179	46.1

Use type of FP (n=209)		
Short acting	163	42
Long-acting	46	11.9

Reason for choosing use FP (n=209)		
By health professional counseling	170	43.8
Perceived from friends experience and safety	27	7
Others	12	3.1

### Child desire to study subjects

**Figure 2:**
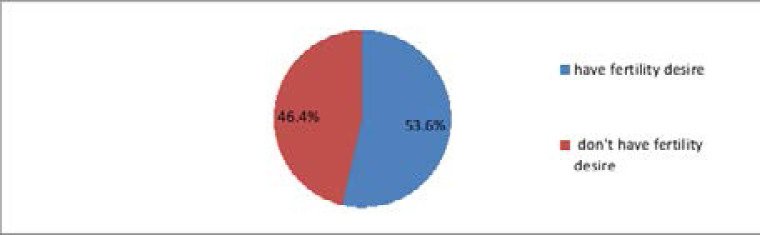
Distribution of Fertility desire of the study among ART clients in Hawassa city southern, Ethiopia, 2019

Fertility desire was assessed by nine questions that compute variables by adding these nine questions with a 1–5 Likert scale after recoding. After compute variables change into categorical variables based on the mean of which below means were don't have fertility desire(no) and above the mean were have fertility desire(yes). Based on these findings people who have fertility desire were 208 (53.6%) whereas 180 (46.4%) people don't have the desire to have a child among the study participants (figure 2).

### Factors associated with fertility desire

Variables that sex, age, sexual practice in the last six months, counseling-RH with ART provider, have a child, knowledge on PMTCT, years since HIV diagnosis, and current relationship were associated with in bivariate analysis. Those variables were re-entered in multivariate analysis for further analysis to identify factors significantly associated with fertility desire. In multivariate analysis age, counseling -RH with ART provider and sexual practices in the last six months were significantly associated with fertility desire (P<0.05). The odds of having fertility desire among these people aged group(18–29) years were 5.7 times higher than those people age group(>=40) years [AOR=5.75,95%CI: (2.85, 11.57)] and the odds of having fertility desire among people age group(30–39) years were 4.7 times as compared to those people age group(>=40) years)[AOR=4.71,95%CI:(2.55, 8.71)]. The odds of fertility desire among those who practiced sex for the last six months were three times those who didn't practice sex in the last six months [AOR=3.00, 95%CI (1.468, 6.169)] and the odds of fertility desire among those who discussed reproductive health with ART providers were 3.1 times higher than those who didn't; discussed it [AOR=3.10, 95%CI, (1.86, 5.15) (Table 3).

**Table 3:** Logistic regression output for fertility desire among clients on ART at Hawassa public health facility Southern, Ethiopia January 2019

Variables		Fertility desire		OR (95%CI)	
Sex		Yes	No	COR (95%CI)	AOR (95%CI)
Male		67(47.9%)	73(52.1%)	0.69 (0.45, 1.05)	0.72[0.44, 1.18]
Female		141 (56.9% )	107(43.1%)	1	
Age	18–29	71(67.6%)	34(32.4%)	6.26(3.32, 11.79)	5.75[2.85,11.57]*
	30–39	115(58.9%)	80(41%)	4.31(2.46, 7.55)	4.71[2.55, 8.71]*
	>=40	22(25%)	66(75%)	1	1
Sexual practice in the last 6 months					
yes		151(58.8%)	106(41.2%)	1.84 (1.209–2.830)	3.00[1.46, 6.16]*
no		57(43.5%)	74(56.5%)	1	1
Current relationship					
Yes		137(60.4%)	90(39.6%)	1.93(1.28, 2.90)	1.11[0.58, 2.10]
no		71(44.1%)	90(55.9%)	1	1
Counseling –RH with ART provider			
yes		109(62.64)	65(37.35)	1.94(1.29,2.93)	3.10[1.86, 5.15]*
no		99(46.26)	115(53.74)	1	1
Have a child					
no		35(64.8%)	19(35.2%)	1.71(0.94, 3.11)	1.79[0.90, 3.52]
Yes		173(51.8%)	161(48.2%)	1	1
Knowledge on PMTCT					
no		108(57.8%	79(42.2%)	1.38(0.92, 2.06)	1.48[0.92, 2.36]
yes		100(49.8%)	101(50.2%)	1	1
Years since HIV diagnosis					
=<3 years		75(66.9%)	45(40.1%)	1.69(1.089,2.628)	1.54[0.94, 2.52]
>=4 years		133(49.6%)	135(50.4%)	1	1

* Reference to significantly associated and 1, reference to constant

## Discussion

The availability of ART and prevention of mother-to-child transmission prong makes great reproductive health issues for people living with HIV. This study assessed the desire for fertility and identifies factors associated with it. The desire of fertility people on ART at public health facilities in Hawassa city showed that 53.6%. This result continues relatively consistent with a study conducted in Addis-Abeba, which was 54.6%, and Harari regional state which contains 56.2%^7^, ^9^ on fertility desire of women in Spain 49%(Victoria Hernando and Mar Masiá 2014). The possible reason might be due to have been HIV patients who want to have their biological child replace themselves, desire to continue their generation, and the value of having a child in the community. Studies had shown that 53.6% continue to be higher than a study conducted in Hosanna southern Ethiopia which have been 36.5% supported by a study at Fnoteselam Hospital northwest Amhara, Horro guduru wolega zone, northwest Oromia, Tigray, and Fitch Hospital Oromia region^10^–^12^,^13^,^14^. It might be due to time difference that increases the quality of life for individuals and better health service providers counseling about sexuality and the possibility of mother to child transmission will decrease. Study in other countries like Brazil, the paternity intention has been 41% 15 and Kenya 45% (Wekesa and Coast 2014), relatively lower than the current study and other African countries Malawi 31% 16 and Tanzania 37.1% 17 also lower than the current study. This might be due to different socio-demographic, socio-economic factors, site, time, and study subjects. Study in Nigeria which obtain78%, and, 22% women and men 18 respectively women had higher fertility desire while comparing to the current study. The diffrences could be explained in terms of variations in socio-demographic characteristics of the populations and cultural concerns of having a big family size. In the multivariate analysis Age, counseling reproductive health with ART providers, and sexual practice in the last six months were significantly associated factors.

Age group (30–39) years of the study subjects that contain 58.9 % and age group (18–29) years that contain 67.6% were to have fertility desire. The younger age group obtain 5.75 times to have more fertility desire than the older age group and the middle age group was 4.71 times to have more fertility desire than the older age group. Younger age was significantly positively associated with a desire for more children among people living with HIV similar study in Jimma 19, Addis Abeba 20, Harare 13, and Afar 13. Other African countries also age continues positively associated with the desire for fertility like Kenya^17^. Other developed nations' age also remains positively associated with factors like the USA 21 and Southern India^22^. It might be due to older age as they have a great probability to achieve their desired number of children and younger age may not achieve the desired number of children or don't have a child at all. Clients who discussion -RH with ART service providers were 3.1 times higher than those compare who didn't have a discussion which has to be a similar study was done at Fnoteselam Hospital^11^. This association showed that ART service providers may be counseling the ART medication had full prevention from mother to child transmission and but not tolled the exact figure of the transmission rate. Other associated factors who performed sexual intercourse in the last six months were 3 times higher fertility desire than those who didn't perform sexual intercourse in the last six months, which Similar study in woliata southern Ethiopia^8^. Other similar studies in participants who have sexual activity in the last six months among clients who did not have sexual activity in the last six months were 24% times lesser to have fertility desire when compared with participants who performed sexual intercourse in the last six months and the rate of unprotected sex contain 69% and 12.5% of women reporting to be pregnant at the time of the study,^13^. This might be due to attempting sexual practice for the success of the desire of a child. The study population largely constituted of people with HIV/AIDS on ART who were enrolled in special counseling groups where they received counseling about the exact figure of transmission rate during pregnancy, laboring, delivery, safe sex, reproductive health knowledge and practice among people on ART. There were sexual behavior-related questions in the study that were sensitive, social desirability bias might have occurred during the process of collecting data, and the possibility of recall bias since most data is based on patients self-report.

## Conclusions

The prevalence of fertility desire in this study was higher than in other studies while factors associated with fertility desire were age, sexual practice in the last six months, and discussing reproductive health with ART providers. Making integrate part of HIV patient care and counseling with reproductive planning the decision and such counseling needs to focus on letting HIV positive people know the risks and methods of prevention while pregnancy.
